# Agonist-dependent internalization and trafficking of the human prostacyclin receptor: A direct role for Rab5a GTPase

**DOI:** 10.1016/j.bbamcr.2008.04.010

**Published:** 2008-10

**Authors:** Martina B. O'Keeffe, Helen M. Reid, B. Therese Kinsella

**Affiliations:** School of Biomolecular and Biomedical Sciences, Conway Institute of Biomolecular and Biomedical Research, University College Dublin, Belfield, Dublin 4, Ireland

**Keywords:** C-tail, carboxyl-terminal tail, CCV, clathrin coated vesicles, FBS, foetal bovine serum, GFP, green fluorescent protein, GPCR, G protein-coupled receptor, GRK, GPCR receptor kinase, HA, hemagglutinin, HEK, human embryonic kidney, hIP, human IP, IP, prostacyclin receptor, Prostacyclin, Receptor, Agonist, Internalization, Rab5, Human

## Abstract

The human prostacyclin receptor (hIP) undergoes rapid agonist-induced internalization by largely unknown mechanism(s). Herein the involvement of Rab5 in regulating cicaprost-induced internalization of the hIP expressed in human embryonic kidney 293 cells was investigated. Over-expression of Rab5a significantly increased agonist-induced hIP internalization. Additionally, the hIP co-localized to Rab5a-containing endocytic vesicles in response to cicaprost stimulation and there was a coincident net translocation of Rab5 from the cytosol/soluble fraction of the cell. Co-immunoprecipitation studies confirmed a direct physical interaction between the hIP and Rab5a that was augmented by cicaprost. Whilst the dominant negative Rab5a^S34N^ did not show decreased interaction with the hIP or fully impair internalization, it prevented hIP sorting to endocytic vesicles. Moreover, the GTPase deficient Rab5a^Q79L^ significantly increased internalization and co-localized with the hIP in enlarged endocytic vesicles. While deletion of the carboxyl terminal (C)-tail domain of the hIP did not inhibit agonist-induced internalization, co-localization or co-immunoprecipitation with Rab5a per se, receptor trafficking was altered suggesting that it contains structural determinant(s) for hIP sorting post Rab5-mediated endocytosis. Taken together, data herein and in endothelial EA.hy 926 cells demonstrate a direct role for Rab5a in agonist-internalization and trafficking of the hIP and increases knowledge of the factors regulating prostacyclin signaling.

## 1 Introduction

The prostanoid prostacyclin, or prostaglandin (PG)I_2_, plays a prominent role in vascular hemostasis, acting as a potent inhibitor of platelet aggregation and as a vasodilator [1]. As one of the major products of cyclooxygenase (COX)-2, it acts as a potent proinflammatory mediator [2,3] and is abundantly produced during cardiac ischemia/reperfusion, offering cytoprotection [4]. Perturbations in the levels of prostacyclin and/or of the prostacyclin receptor (IP) have been associated with a host of pathologies including thrombosis, stroke, ischemic heart disease, systemic and pregnancy-induced hypertension [5–7]. The critical role of prostacyclin to vascular integrity has been highlighted through findings that certain COXIBs, the subclass of nonsteroidal anti-inflammatory drugs designed to selectively inhibit COX-2, depress prostacyclin generation predisposing patients to increased risk of thrombotic stroke and myocardial infarction [8].

As a member of the G protein coupled receptor (GPCR) superfamily, the prostacyclin receptor (IP) is primarily coupled to Gs/adenylyl cyclase activation but may also couple to a range of other secondary effectors in a cell and/or species specific manner [9–14]. The IP is subject to a host of post-translational modifications that play a central role in regulating its function. For example, the IP is somewhat unique among members of the GPCR superfamily in that it is subject to both isoprenylation and palmitoylation, lipid modifications that occur within its carboxyl-terminal (C)-tail domain and which collectively modulate its G-protein coupling and effector signaling [15–17].

A common feature of GPCR signaling is the regulation or desensitization of second messenger generation and signaling that occurs in response to the sustained agonist stimulation, dampening or terminating the specific cellular response [18,19]. Such desensitization is typically initiated by GPCR phosphorylation, uncoupling the receptor from its cognate G-protein, and may lead to sequestration and internalization of the desensitized GPCR from the plasma membrane into intracellular compartments [18,19]. Whilst several studies have established that the IP undergoes rapid agonist-induced phosphorylation, internalization and down-regulation, such as in human platelets and other cell types to critically modulate prostacyclin responses, the mechanism(s) by which the hIP undergoes such internalization remains largely undetermined [20–24]. The classic mechanism by which GPCRs undergo agonist-induced (homologous) desensitization is initiated by G-protein-coupled receptor kinase (GRK)-mediated phosphorylation. Thereafter, β-arrestin is recruited to the GRK-phosphorylated receptor and acts as an adapter to target GPCRs to clathrin coated pits by means of their association with both clathrin and the β2-adaptin subunit of the heterotetrameric AP2 adaptor complex [18,19]. Dynamin mediates the first steps of endosome formation at sites of clathrin-coated vesicles (CCVs) and caveoli [25,26]. Rab proteins, the largest subgroup of the Ras superfamily of GTPases, are involved in the regulation of vesicular protein transport in endocytosis, trafficking, endosome fusion and exocytosis with each Rab having a distinct cellular localization and role in the transport process [27–30]. Rab5 is localized to the plasma membrane, CCVs and early endosomes [31] and has been shown to be involved in the internalization of many GPCRs including the β_2_ adrenergic receptor [32,33], endothelin A and B receptors [34], the D2 dopamine receptor [35], the neurokinin-1 receptor [36] and the angiotensin II type 1A receptor [37].

Whilst the hIP undergoes direct agonist-induced PKC phosphorylation within its C-tail domain, desensitizing its signaling [23], agonist-induced internalization of the hIP is completely independent of PKC [24]. Moreover, while it was also established that agonist-induced internalization of the hIP does not occur through the classic GRK/β-arrestin mechanism [24], the internalized receptor was confirmed to co-localize to CCVs and internalization was impaired, at least in part, by inhibitors of clathrin-mediated trafficking and of dynamin signaling [24]. The carboxyl-terminal (C)-tail domain of a given GPCR can act as an important determinant of receptor desensitization and internalization, frequently enriched in Ser/Thr residues for phosphorylation and containing structural motifs for interaction with components of various trafficking paths, such as β-arrestin or indeed Rab protein binding domains [18,19,37,38]. In the case of the hIP, there is considerable controversy in the literature regarding the role of its C-tail domain in its agonist-induced internalization [16,24,39,40]. Thus, in view of the critical importance for the dynamic regulation of the cellular responses to prostacyclin, such as within the vasculature, coupled to the apparent controversy surrounding the actual mode of regulation of those responses through IP internalization post-signaling, the aim of the current study was to investigate the mechanisms of agonist-induced internalization of the hIP and determine a role, if any, for the Rab5 GTPase in that internalization. Our data established that the hIP is internalized in an agonist-dependent manner into Rab5a-containing endosomes through a mechanism that promoted its sorting into enlarged endocytic vesicles and involves a direct physical interaction between the hIP and Rab5a.

## 2 Experimental procedures

### 2.1 Materials

Cicaprost was obtained from Schering AG (Berlin, Germany). [^3^H]iloprost was purchased from Amersham Biosciences. Mouse monoclonal anti-hemagglutinin (HA)-101R antibody (1 mg/ml) was obtained from Cambridge Biosciences; rabbit polyclonal anti-Rab5 (S-19), rabbit polyclonal anti-GFP (FL), horse radish peroxidase (HRP)-conjugated goat anti-rabbit and HRP-conjugated goat anti-mouse (400 μg/ml) secondary antibodies were from Santa Cruz; rat monoclonal anti-HA 3F10-HRP-conjugated antibody (25 μg/ml) was obtained from Roche. Anti HDJ-2 antibody was from Neomarkers. AlexaFluor594 goat anti-mouse (2 mg/ml) and AlexaFluor488 goat anti-rabbit (2 mg/ml) antibodies were from Molecular Probes. DAPI and Protein-G-Sepharose were obtained from Sigma. The plasmid pcDNA3:HA:Dynamin^K44A^ was kindly provided by Dr J. Benovic, Thomas Jefferson University, PA, USA.

### 2.2 Site-directed mutagenesis and subcloning

The plasmid pCMV5:Rab5a has been previously described [41]. Conversion of Ser^34^ to Asn^34^ of Rab5 to generate pCMV5:Rab5a^S34N^ was achieved using pCMV5:Rab5a as template and the sense/antisense primer pair (5′ G TCC GCT GTT GGC AAA **AAT** AGC CTA GTG CTT CG). Conversion of Gln^79^ to Leu^79^ to generate pCMV5:Rab5a^Q79L^ was achieved using pCMV5:Rab5a as template and the sense/antisense primer pair (5′ GG GAT ACA GCT GGT **CTT** GAA CGA TAC CAT AGC C). Sequences shown correspond to the sense primer and the identity of the mutator codon is in boldface italics. All site directed mutagenesis was performed using QuikChange™ (Stratagene) system and were validated by DNA sequence analysis. The full length cDNAs encoding Rab5a, Rab5a^S34N^, Rab5a^Q79L^ were subcloned in frame into the Xho1-BamH1 sites of pEGFPC1 to generate pEGFPC1:Rab5a, pEGFPC1:Rab5a^S34N^ and pEGFPC1:Rab5a^Q79L^, respectively.

### 2.3 Cell culture and transfections

Human embryonic kidney (HEK) 293 cells were obtained from the American Type Culture Collection and were grown in minimal essential medium (MEM) containing 10% foetal bovine serum (FBS).

Routinely approximately 48 h prior to transfection, HEK293 cells were plated at a density of 2 × 10^6^ cells/10 cm culture dish in 8 ml media. Thereafter, cells were transiently transfected with 10 μg of pADVA [42] and 25 μg of pCMV- or pEGFPCI-based vectors using the calcium phosphate/DNA co-precipitation procedure, as previously described [43]. Transiently transfected cells were harvested 48 h after transfection, unless otherwise stated.

HEK.hIP^WT^, HEK.hIP^Δ312^ and HEK.hIP^Δ307^ cells stably over-expressing HA-tagged forms of hIP^WT^, hIP^Δ312^ and hIP^Δ307^, respectively, have been previously described [16,17]. HEK.β-galactosidase (HEK.β-Gal) cells stably over-expressing HA-tagged β-galactosidase (β-Gal) from *Escherichia coli* were generated essentially as previously described [15].

EA.hy 926 cells were obtained from the Tissue Culture Facility at UNC Lineberger Comprehensive Cancer Center, North Carolina and were grown in Dulbecco's modified Eagle's medium (DMEM) containing 10% FBS [44].

### 2.4 Radioligand binding studies

HEK.hIP, HEK. hIP^Δ312^, HEK.hIP^Δ307^ cells were harvested by centrifugation at 500 ×*g* at 4 °C for 5 min and washed three times with phosphate-buffered saline (PBS). For membrane preparation, cells were resuspended in homogenization buffer (25 mM Tris–HCl, pH 7.5, 0.25 M sucrose, 10 mM MgCl_2_, 1 mM EDTA, 0.1 mM phenylmethylsulfonyl fluoride), and membrane fractions were prepared by homogenization followed by centrifugation (100,000 ×*g*, 40 min at 4 °C). The pellet fraction (P_100_), representing crude membranes, was resuspended in MES-KOH buffer (10 mM MES-KOH, pH 6.0, 10 mM MnCl_2_, 1 mM EDTA, 10 mM indomethacin). IP radioligand binding assays were carried out at 30 °C for 1 h using 100 µg of membrane protein (P_100_) in 100 µl reactions in the presence of 4 nM [^3^H]iloprost (15.3 Ci/mmol) for saturation binding studies, or in the presence of 0.1 nM–200 nM [^3^H] iloprost for Scatchard analysis, as described previously [16,17].

### 2.5 Internalization of human prostayclin receptor (hIP) through ELISA

Cells were seeded at a density of 5 × 10^4^ cells/ml per well in 1 ml of MEM, 10% FBS media into 24-well plates, pre-coated with 0.001% poly-l-lysine, and were grown for 48 h at 37 °C prior to experiments. To assess agonist-induced internalization of hIP or its mutated derivatives, the media was changed to serum-free MEM and thereafter, cells were treated with cicaprost (1 μM, or for dose response, 0–10 μM) for 0, 0.5, 1, 1.5, 2, 3 and 4 h at 37 °C. Cells were washed twice in ice-cold PBS prior to fixation in 3.7% paraformaldehyde, TBS, pH 7.4, for 5 min at room temperature. After washing the cells three times in TBS (20 mM Tris–HCl, pH 7.4, 0.1 M NaCl), non-specific sites were blocked with Blocking Buffer (1% bovine serum albumin, BSA, in TBS) for 1 h at room temperature. Thereafter, cells were incubated with anti-HA 101R antibody (1: 2000 in Blocking Buffer) for 1 h at room temperature. The antibody solution was removed and the cells were washed three times in TBS, prior to incubation with goat anti-mouse HRP (1: 2000) for 1 h at room temperature. Following this, the cells were washed three times in TBS and net changes in HA-tagged receptor cell surface expression were determined colorimetrically at 650 nm using the K-Blue substrate (Neogen Corp) as previously described [16].

For internalizations involving the co-expression of the various Rab proteins, HEK.hIP, HEK. hIP^Δ312^, HEK.hIP^Δ307^ or, as controls, HEK 293 cells (10 cm dishes, 60–70% confluent) were transiently transfected with 10 μg of pADVA (Gorman and McCray, 1990) and 25 μg of pCMV:Rab5a, pCMV5:Rab5a^S34N^, pCMV5:Rab5a^Q79^, pcDNA3:dynamin^K44A^ or, as controls, the empty vector equivalents using the calcium phosphate/DNA co-precipitation procedure, as previously described [43]. Some 24 h post-transfection, cells were transferred to poly-l-lysine (0.001%) pre-coated 24-well plates, at a density of 5 × 10^4^ cells per well. Following 48 h incubation at 37 °C, cells were stimulated as indicated in the figure legends and the ELISA assay carried out to determine levels of cell surface expression of HA-tagged hIPs, as previously described.

### 2.6 Indirect immunofluorescence microscopy

To monitor expression of the hIP by indirect immunofluorescence, HEK.hIP cells were grown to 60–70% confluency on poly-l-lysine pre-treated coverslips in 6-well plates. Thereafter, cells were washed in serum-free medium (MEM) and incubated with either vehicle (MEM) or 1 μM cicaprost, in MEM, for 2 h at 37 °C. Cells were washed twice in ice-cold PBS prior to fixation in 3.7% paraformaldehyde, PBS, pH 7.4, for 15 min at room temperature. After washing three times in PBS, cells to be permeabilized were incubated with 0.2% Triton X-100 in PBS for 10 min on ice followed by washing in three times in TBS. Non-specific sites were blocked by incubating cells with Blocking Buffer (1% BSA in TBS) prior to immunolabeling with anti-HA 101R (1:1000 dilution in Blocking Buffer) for 1 h. Unbound antibody was then removed by washing twice in TBS followed by detection with the secondary AlexaFluor594 goat anti-mouse antibody (1:5000, in 1% BSA, TBS). Cells were washed three times in TBS, prior to counterstaining with DAPI (1 μg/ml, in H_2_O). Excess DAPI was washed away with H_2_O prior to mounting coverslips in DakoCytomation fluorescence mounting medium.

### 2.7 Assessment of agonist-induced internalization by confocal microscopy

In order to monitor changes in cell-surface expression of the hIP as a function of cicaprost-stimulation and/or colocalization with Rab5, HEK.hIP cells were transiently transfected with 10 μg of pADVA and 25 μg of pEGFPC1:Rab5a, pEGFPC1:Rab5a^S34N^, pEGFPC1:Rab5a^Q79L^, encoding green fluorescent protein (GFP)-tagged forms of the Rab proteins using the calcium phosphate/DNA co-precipitation procedure [43]. Some 24 h later, cells were seeded onto poly-l-lysine pre-treated coverslips in 6-well plates to achieve 60–70% confluency following 48 h incubation at 37 °C. Thereafter, the cells were washed in serum-free MEM and then pre-incubated with anti-HA 101R (1:1000 dilution in MEM) at 4 °C for 1 h to label cell surface receptors. Unbound antibody was removed by washing twice with MEM following which cells were either analysed immediately (0 h) or were incubated with 1 μM cicaprost in MEM for 0–4 h, as indicated, at 37 °C. Cells were then washed twice in ice-cold PBS prior to fixation and permeabilization, as described above. Non-specific sites were blocked and HA-tagged receptors were immunolabelled with the secondary AlexaFluor594 goat anti-mouse antibody, as described. In order to examine cicaprost-dependent changes in hIP cell-surface expression and/or colocalization with endogenous Rab5, untransfected HEK.hIP cells were preimmunolabeled with anti-HA 101R (1:1000 dilution in MEM; 4 °C for 1 h) prior to agonist stimulation (1 μM cicaprost; 0–4 h) as described above. After fixation and permeabilization, non-specific sites were blocked followed by immunolabelling of endogenous Rab5 with anti-Rab5 (S-19). Thereafter, HA-tagged receptors and endogenous Rab5 were immunolabelled with the secondary AlexaFluor594 goat anti-mouse and AlexaFluor488 goat anti-rabbit antibodies, respectively, as described. Similarly, EA.hy 926 cells were treated with 1 μM cicaprost prior to fixation, permeabilization and immunolabelling with anti-Rab5 and secondary AlexaFluor488 goat anti-rabbit antibodies. All slides were imaged, at ×63 magnification, using Carl Zeiss Lazer Scanning System LSM510 and Zeiss LSM Imaging software for acquiring multichannel images with filters appropriate for enhanced GFP, AlexaFluor488 and AlexaFluor 594 fluorescence.

### 2.8 Subcellular fractionation

HEK.hIP cells (approx 2 × 10^6^ cells in 8 ml MEM, 10% FBS) were plated on 10-cm dishes some 48 h previously to achieve approximately 75% confluency. Thereafter, cells were washed and incubated in serum-free MEM for 1 h prior to treatment with 1 μM cicaprost for 0, 0.5, 1, 2, 3 or 4 h. While retaining an aliquot of total protein for SDS-PAGE analysis, subcellular fractionation was carried out on the remaining sample. Briefly, cell pellets were resuspended in homogenization buffer (25 mM Tris–Cl, pH 7.5, 0.25 M sucrose, 10 mM MgCl_2_, 1 mM EDTA, 0.1 M PMSF) and homogenized on ice for 1 min (approximately 20 strokes) prior to centrifuging samples at 100,000 ×*g* for 60 min at 4 °C. The soluble, supernatant (S_100_ fraction) was retained for analysis and the pellet (P_100_ fraction) was first washed in MES-KOH buffer (10 mM MES-KOH, pH 6.0, 10 mM MnCl_2_, 1 mM EDTA, 10 mM indomethacin) prior to resuspension in 10 mM Tris–Cl, 1 mM EDTA., pH 8.0. The protein concentrations were determined by the Bradford assay. Aliquots of the Total, S_100_ and P_100_ protein fractions (50 μg per lane) were resolved by SDS-PAGE, on 12.5% gels, and subject to immunoblotting with anti-Rab5 antibody (1:1000) followed by anti-HDJ-2 (1:4000) antibody, with chemiluminescence detection [15]. To quantify changes in Rab5 expression in the S_100_ fractions as a function of cicaprost stimulation (h), all images of Rab5 expression in the S_100_ fractions were captured using Adobe Photoshop (V6), where band width and intensity was quantified. Thereafter, Rab5 expression in the S_100_ fractions at the various time points was expressed as a percentage of that in the absence of cicaprost (Rab5 Expression in S_100_; % Expression ± S.E.M., *n* = 3).

### 2.9 Co-Immunoprecipitations

HEK.hIP^wt^, HEK.hIP^Δ312^, HEK.hIP^Δ307^and, as controls, HEK.293 or HEK.β-galactosidase (β-Gal) cells, were transiently co-transfected with pADVA and either pEGFPC1, pEGFPC1:Rab5a, pEGFPC1:Rab5a^S34N^ or pEGFPC1:Rab5a^Q79L^, using the calcium phosphate/DNA co-precipitation procedure [43]. Some 48 h post-transfection, cells were washed in serum-free MEM and either incubated with vehicle (serum-free MEM) or with 1 μM cicaprost in serum-free MEM for 2 h at 37 °C, or as indicated in the figure legends. Thereafter, the incubation was stopped by washing the cells twice in ice-cold PBS followed by lysis in 500 μl radio-immune precipitation buffer (RIP; 50 mM Tris HCl, pH 8.0, 150 mM NaCl, 1 mM EDTA, 1% Nonidet P-40 (v/v), 0.5% sodium deoxycholate (w/v), 0.1% SDS (w/v), 10 mM sodium fluoride, 25 mM sodium pyrophosphate, 1 mM PMSF, 4 µg/ml leupeptin, 2.5 µg/ml aprotinin). Lysates were clarified by centrifugation at 13,000 rpm for 5 min and 50 μl (approx 50 μg) retained for analysis of protein expression in whole cell lysates. The remaining lysate was used for immunoprecipitation, using anti-HA 101R antibody (1:300) to pull-down HA-tagged hIP(s) through overnight incubation at 4 °C with mixing on a rotissary. Thereafter, the lysates were incubated for 1 h with 10 µl of 50% slurry of protein G Sepharose, prior to washing. Immunoprecipitates were resolved by SDS-PAGE, on 10% gels, and subjected to successive immunoblotting with anti-Rab5 (1:1000), anti-GFP (1:1000) and anti-HA 3F10-HRP (1:500) antibodies.

### 2.10 Data analyses

Statistical analyses were carried out using the unpaired Student's *t* test throughout or, where relevant and specifically indicated in text, using two-way ANOVA employing the GraphPad Prism (version 4.00) package. *p*-values of less than or equal to 0.05 were considered to indicate a statistically significant difference.

## 3 Results

### 3.1 Effect of Rab5 on agonist-induced internalization of the human prostacyclin receptor, hIP

**Fig. 1:**
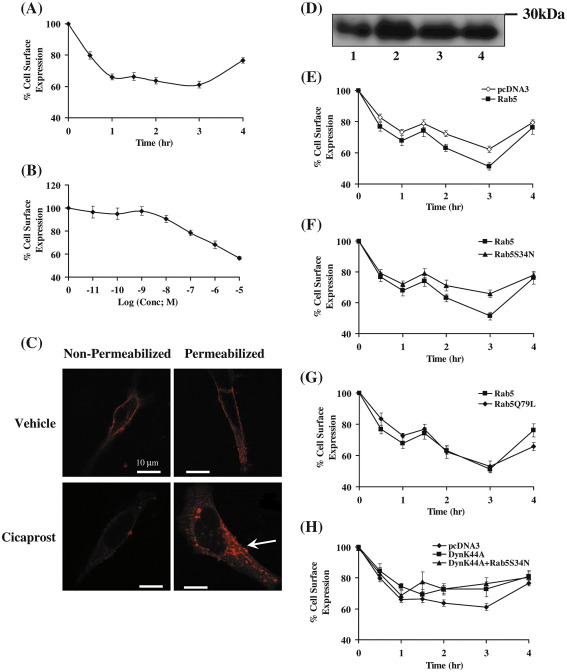
Cicaprost-induced Internalization of the human Prostacyclin Receptor (hIP). Panels A and B: HEK.hIP cells were incubated at 37 °C with 1 μM cicaprost for 0–4 h (panel A) or with 0–10 μM cicaprost for 4 h (panel B). Panels E–G: HEK.hIP cells transiently transfected with either the control vector pcDNA3 (panel E), pCMV5:Rab5a (panels E–G), pCMV5:Rab5a^S34N^ (panel F) or pCMV5:Rab5a^Q79L^ (panel G) were incubated with 1 μM cicaprost at 37 °C for 0–4 h. Panel H, HEK.hIP cells, transiently transfected with pcDNA3:dynamin^K44A^, pCMV5:Rab5a plus pcDNA3:Dynamin^K44A^ or with pcDNA3 were incubated at 37 °C with 1 μM cicaprost for 0–4 h. Panels A, B, E–H: expression of cell surface HA-tagged hIPs was detected by ELISA using anti-HA 101R antibody. Results are either expressed as mean cell surface expression as a percentage of that at 0 h (% cell surface expression ± S.E.M.) as a function of time (h) or of Log_10_ cicaprost concentration (M; panel B) and, in each case, are representative of at least three independent experiments each carried out in triplicate. Panel C: HEK.hIP cells were preincubated with the drug vehicle (vehicle) or with 1 μM cicaprost for 2 h at 37 °C (Cicaprost); thereafter cells were immunolabelled using anti-HA 101R antibody under either non-permeabilizing and permeabilizing conditions. Images (*n* = 3) were captured using Carl Zeiss Lazer Scanning System LSM510 and Zeiss LSM Imaging software. The horizontal bar represents 10 μm. Panel D: HEK.hIP cells transiently transfected with either pcDNA3 (lane 1), pCMV5:Rab5a (lane 2), pCMV5:Rab5a^S34N^ (lane 3) or pCMV5:Rab5a^Q79L^ (lane 4) were analyzed by SDS-PAGE (50 μg whole cell protein per lane) followed by immunoblotting using anti-Rab5 antibody. Data presented is a representative immunoblot from 3 independent experiments. The reader is referred to the web version of this article to see color images of this figure, where relevant.

Whilst the human prostacyclin receptor (hIP) undergoes rapid agonist-induced phosphorylation and desensitization of signaling, it has also been widely reported to undergo agonist-induced internalization through, as yet, largely unknown mechanism(s). Hence, we sought to elucidate the mechanism of agonist-induced internalization of the hIP stably expressed in human embryonic kidney (HEK) 293 cells in response to its selective agonist cicaprost. To begin with, we used an ELISA-based internalization assay to measure net changes in cell surface expression of hemagglutinin (HA)-tagged hIPs in response to cicaprost stimulation of HEK.hIP cells, a previously characterized clonal cell line [15,17]. The hIP underwent cicaprost-induced internalization in a biphasic manner, with rapid internalization observed during the first 60 min and, thereafter, reached a plateau or reduced rate up to 3 h such that, in all, approximately 40% of cell surface hIP underwent internalization following 3 h stimulation (61.2 ± 2.15% cell surface expression; Fig. 1A). Thereafter, the overall level of hIP expression at the cell surface increased significantly with some 76.6 ± 1.76% expressed on the cell surface at 4 h post cicaprost stimulation, suggesting that approximately 50% of the internalized hIP may recycle back to the cell surface with time. The inclusion of the general protein synthesis inhibitor cycloheximide did not affect the overall pattern or profile of cicaprost-induced internalization by the hIP, confirming that the increased cell surface expression observed at 4 h was due to receptor recycling or trafficking from intracellular pools and not due to de novo protein synthesis (data not shown). Internalization was also concentration dependent with 2 × 10^− 7^ M cicaprost required to bring about a 20% decrease in hIP expression on the cell surface (i.e. concentration required to induce internalization of 50% of internalizable hIPs; Fig. 1B). Furthermore, immunofluorescence and confocal imaging showed that in resting HEK.hIP cells, the hIP is predominantly expressed on the cell surface as demonstrated in both non-permeabilized and permeabilized cells, with evidence from the latter that some of the hIP is also expressed intracellularly (Fig. 1C, upper panels). Stimulation of HEK.hIP cells with 1 μM cicaprost (2 h at 37 °C) led to a significant decrease in the expression of the hIP detected at the cell surface under non-permeabilizing conditions (Fig. 1C). Concomitant with this, under permeabilizing conditions, there was a substantial increase in the intracellular expression of the hIP, notably to large punctate vesicular-type structures reminiscent of clathrin-coated vesicles (CCV) and early endosomes (Fig. 1C, lower panels).

While the most prevalent, and indeed best characterized, pathway by which GPCRs internalize in response to agonist-stimulation is via the classic GRK-mediated phosphorylation/β-arrestin-dependent mechanism leading to internalization on clathrin-coated and non-clathrin coated vesicles, as stated, agonist-induced internalization of the hIP is independent of the GRKs/β-arrestins but is dependent on clathrin and, partially, dependent on dynamin [24]. The GTPase Rab5 is also widely reported to participate in the internalization and/or recruitment of numerous GPCRs to clathrin-coated, Rab5 containing early endosomes [33–36]. Therefore, in view of the significant agonist-induced relocalization of the hIP away from the plasma membrane to the large intracellular vesicular or endosomal structures, as was evident from our immunolocalization data (Fig. 1C), we investigated the possible involvement of Rab5 in the internalization of the hIP. Hence, initially herein, the effect of over-expression of Rab5a on agonist-induced internalization of the hIP was examined using an ELISA-based internalization assay to monitor over-all changes in cell-surface hIP. The expression of significant levels of both endogenous Rab5 and increased expression of recombinant Rab5a in HEK.hIP cells (Fig. 1D) and indeed in the parental HEK 293 cell line (data not shown) was initially confirmed by western blot analysis. While over-expression of Rab5a did not affect the initial rate or overall biphasic pattern of cicaprost-induced internalization of the hIP in HEK.hIP cells relative to that in non-transfected (Fig. 1A) or cells transfected with the empty control vector pcDNA3 (Fig. 1E), it significantly increased the overall level of hIP internalization (Fig. 1E, *p* < 0.0001; two-way ANOVA), with most significance at 2 and 3 h (Fig. 1E; *p* = 0.005 and *p* = 0.0076, respectively). Moreover, while over-expression of a dominant negative form of Rab5a, namely Rab5a^S34N^, did not appear to affect the initial rate or overall biphasic profile of cicaprost-induced hIP internalization over the 4 h incubation period, it significantly impaired hIP internalization relative to that of the wild type Rab5a (Fig. 1F; *p* = 0.0003; ANOVA), with most significance at 2 and 3 h (Fig. 1E; *p* = 0.05 and *p* = 0.0001, respectively). The fact that there is such abundant expression of endogenous Rab5 in HEK.hIP cells may explain why Rab5a^S34N^ did not result in a more substantial inhibition of internalization of the hIP (Fig. 1F). Conversely over-expression of the constitutively active Rab5a^Q79L^ led to significantly increased cicaprost-induced hIP internalization compared to non-transfected or control cells transfected with pcDNA (*p* = 0.0005; ANOVA), but Rab5a^Q79L^ did not alter the overall profile or extent of that hIP internalization relative to the wild type Rab5 (Fig. 1G, *p* = 0.6718). However, Rab5a^Q79L^ did appear to impair recycling of the hIP to the plasma membrane at the 4 h time point (Fig. 1G; *p* = 0.039).

In a previous study, Smyth et al., established that iloprost-mediated internalization of hIP was also partially reduced by over-expression of a dominant negative form of dynamin, namely Dyn^K44A^ [24]. Hence, in view of our findings herein involving Rab5, we sought to determine whether expression of Dyn^K44A^ alone or Dyn^K44A^ along with Rab5a^S34N^ might affect the overall level of cicaprost-induced hIP internalization. Over-expression of Dyn^K44A^ and Rab5a^S34N^ in HEK.hIP^WT^ cells was confirmed by western blot analysis (data not shown). Moreover, expression of Dyn^K44A^ significantly decreased the extent of cicaprost-induced internalization of the hIP relative to control (pcDNA)-transfected cells (Fig. 1H; *p* < 0.0001; ANOVA). Similarly, while co-expression of both Dyn^K44A^ along with Rab5a^S34N^ impaired cicaprost-induced internalization in HEK.hIP^WT^ cells relative to pcDNA-transfected cells (Fig. 1H, *p* < 0.0001; ANOVA), co-expression of both factors together did not significantly impair internalization relative to Rab5a^S34N^ alone (Fig. 1F, *p* = 0.27; ANOVA) or Dyn^K44A^ alone (Fig. 1H, *p* = 0.8068; ANOVA). Hence, the lack of an accumulative effect of Dyn^K44A^ and Rab5a^S34N^ on inhibition of agonist-induced internalization of the hIP, suggests that dynamin and Rab5a regulate that internalization through a common pathway rather than through 2 separate or independent pathways.

### 3.2 Agonist-induced colocalization of internalized hIP to Rab5 endosomes

**Fig. 2:**
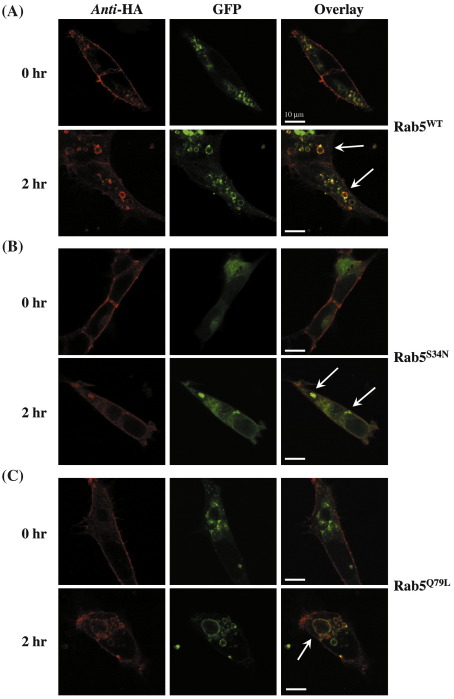
Cicaprost-induced Co-localization of the hIP and Rab5a, Rab5a^S34N^ or Rab5a^Q79L^. HEK.hIP cells, transiently transfected with either pEGFPC1:Rab5a (panel A), pEGFPC1:Rab5a^S34N^ (panel B), pEGFPC1:Rab5a^Q79L^ (panel C) were pre-labelled with anti-HA 101R primary antibody for 1 h at 4 °C; thereafter, cells were either analyzed directly (0 h) or were incubated at 37 °C for 2 h with 1 μM cicaprost (2 h), as indicated. Cells were fixed and permeabilized prior to detection of HA-tagged hIPs, with anti-mouse AlexaFluor594 conjugated secondary antibody, and enhanced GFP:Rab5 expression using a Carl Zeiss Lazer Scanning System LSM510 and Zeiss LSM Imaging software. Data presented are representative images from 3 independent experiments from which at least 10 fields were viewed at ×63 magnification, where the horizontal bar represents 10 μm. The reader is referred to the web version of this article to see color images of this figure.

To further assess the role of Rab5, HEK.hIP cells were transiently co-transfected with plasmids encoding green fluorescent protein (GFP)-tagged forms of Rab5a, Rab5a^S34N^ or Rab5a^Q79L^ and cicaprost-induced internalization of the cell-surface of HA-tagged hIP and possible co-localization with Rab5a examined by confocal microscopy. Specifically, cell surface HA-tagged receptors in HEK.hIP cells were initially pre-immunolabelled at 4 °C with the anti-HA 101R antibody; thereafter, HA-hIP expression was either analyzed directly (0 h internalization) or following incubation of cells with 1 μM cicaprost for 2 h at 37 °C (2 h post-agonist stimulation). In the absence of agonist stimulation, immunolabelled HA-hIPs were expressed at the cell surface as expected whereas GFP-Rab5a was localized to distinct intracellular vesicular structures, with little to no co-localization of the hIP and Rab5a evident (Fig. 2A, Rab5^WT^, 0 h). Following stimulation of cells with cicaprost for 2 h, there was profound relocalization of the hIP away from the cell surface to enlarged vesicular, Rab5a positive endosomes with significant co-localization evident in the over-laid images (Fig. 2A, Rab5^WT^, 2 h). Hence, these data indicate that the hIP located at the cell surface internalizes to intracellular Rab5-positive endosomes in response to cicaprost stimulation. However, the experimental approach of pre-immunolabelling the hIPs expressed at the cell surface does not examine the agonist-dependent colocalization or association of any pre-existing intracellular pools of the hIP with Rab5.

In cells over-expressing the dominant negative Rab5a^S34N^, the pre-immunolabelled hIP was exclusively expressed at the cell surface, as expected, in the absence of agonist stimulation while GFP-Rab5a^S34N^ exhibited diffuse intracellular staining associated with significantly smaller vesicles than those associated with the Rab5a^WT^ (Figs. 2A and B, GFP). Whilst incubation of the cells over-expressing Rab5a^S34N^ with cicaprost for 2 h led to internalization of the hIP, consistent with the ELISA-based internalization studies the extent of relocalization of the hIP away from the plasma membrane was significantly impaired and the pattern of intracellular staining was more diffuse, lacking the distinct vesicular pattern observed in the HEK.hIP cells expressing the wild type Rab5a (Fig. 2B, Anti-HA). Upon agonist stimulation, the Rab5a^S34N^ did coalesce into more punctate structures but they were not as numerous nor did they exhibit clearly defined ring-like vesicular structures, as per the wild type Rab5a. In the overlaid image there was some evidence of co-localization, as indicated by the arrows, but the majority of the hIP did not co-localize with Rab5a^S34N^ (Fig. 2B, overlay). In the absence of agonist, the GTPase-defective Rab5a^Q79L^ primarily localized to pre-formed enlarged vesicular endosomes and showed no significant co-localization with the hIP expressed on the cell surface (Fig. 2C). Furthermore, stimulation of cells with cicaprost led to a significant increase in the size of the Rab5a^Q79L^-positive endosomal structures (Fig. 2C, GFP). Concomitant with this, pre-immunolabelled cell surface hIP internalized in response to cicaprost stimulation into intracellular vesicular endosomes that were markedly increased in size compared to those in cells over-expressing Rab5a^wt^ (Fig. 2C, Anti-HA). In the overlaid images, it was evident that the internalized hIP almost exclusively co-localized with the GFP-Rab5a^Q79L^ in those enlarged endosomes (Fig. 2C, overlay).

**Fig. 3:**
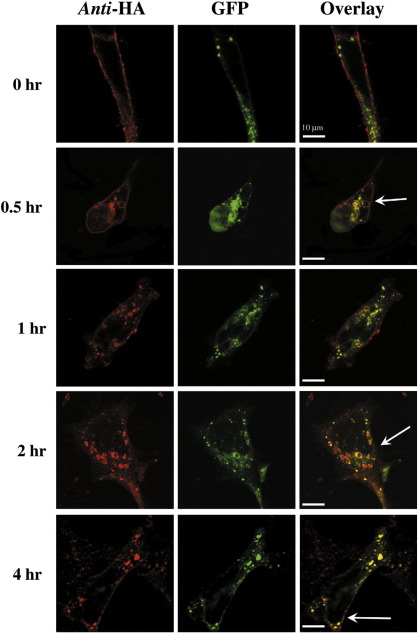
Cicaprost-induced Co-localization of the hIP and Rab5a. HEK.hIP cells, transiently transfected with the pEGFPC1:Rab5a, were pre-labelled with the anti-HA 101R antibody for 1 h at 4 °C, prior to stimulation with vehicle or 1 μM cicaprost at 37 °C for 0–4 h. Cells were fixed and permeabilized prior to detection of HA-tagged hIPs, with anti-mouse AlexaFluor594 conjugated secondary antibody, and enhanced GFP:Rab5a expression using Carl Zeiss Lazer Scanning System LSM510 and Zeiss LSM Imaging software. Data presented are representative images from 3 independent experiments from which at least 10 fields were viewed at ×63 magnification, where the horizontal bar represents 10 μm. The reader is referred to the web version of this article to see color images of this figure.

**Fig. 4:**
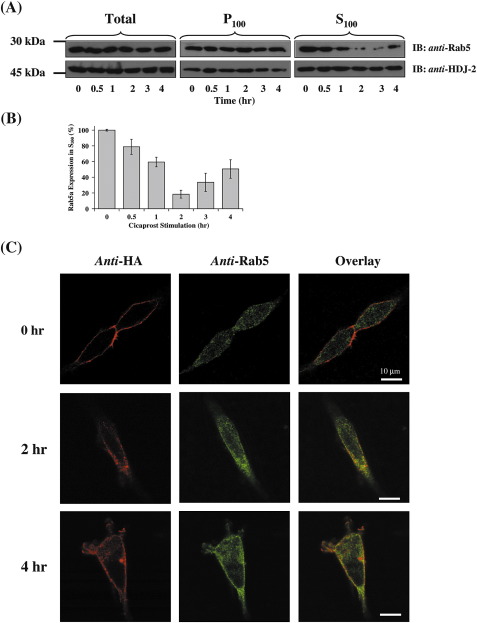
Cicaprost-induced Sub-cellular Translocation of Rab5. Panel A: HEK.hIP cells were incubated with 1 μM cicaprost for 0, 30 min, 1, 2, 3 and 4 h at 37 °C in serum-free media prior to homogenization and subcellular fractionation into their respective particulate (P_100_) or soluble (S_100_) fractions following centrifugation at 100,000 ×*g* for 1 h at 4 °C. Aliquots (50 μg protein per lane) of the resulting total, P_100_ and S_100_ protein fractions were resolved by SDS-PAGE and initially immunoblotted (IB) with anti-Rab5 antibody (upper panels); followed by anti-HDJ-2 antibody (lower panels). Results presented are representative from 3 independent experiments. The relative positions of the 30 kDa and 45 kDa molecular size markers are indicated to the left of the panels. Panel B: Rab5 expression in the S_100_ fractions at the various time points following cicaprost stimulation (h) as a percentage of that in the absence of cicaprost (Rab5 expression in S_100_; % Expression ± S.E.M.). Panel C: HEK.hIP cells were pre-labelled with the anti-HA 101R antibody for 1 h at 4 °C, prior to stimulation with vehicle or 1 μM cicaprost at 37 °C for 0–4 h. Cells were fixed and permeabilized prior to detection of HA-tagged hIPs, with anti-mouse AlexaFluor594 conjugated secondary antibody, and endogenous Rab5 with anti-Rab5 (S-19) and anti-rabbit AlexaFluor488 conjugated secondary antibody, using Carl Zeiss Lazer Scanning System LSM510 and Zeiss LSM Imaging software. Data presented are representative images from 3 independent experiments from which at least 10 fields were viewed at ×63 magnification, where the horizontal bar represents 10 μm. The reader is referred to the web version of this article to see color images of this figure.

To further examine the role of Rab5a in agonist-induced internalization of hIP, we next investigated the time-dependent internalization and co-localization of the hIP to Rab5a positive endosomes over a 4 h time course (Fig. 3). As in the previous experiment, cell surface HA-tagged hIPs were pre-immunolabelled prior to stimulation with cicaprost to follow their internalization into the Rab5a positive endosomes. In the absence of agonist, the pre-labelled hIP was detected exclusively at the cell surface while, in response to cicaprost stimulation it quickly re-localized away from the plasma membrane and internalized to enlarged Rab5a positive endosomes in a time-dependent manner (Fig. 3, Anti-HA). Even at 30 min post-agonist stimulation, there was evidence that the hIP and Rab5a co-localized to the cell surface and to vesicles just inside the cell membrane (Fig. 3, 0.5 h; indicated by the arrows) while at 1 h–2 h post-stimulation, there was almost complete loss of cell surface receptors and increased association with Rab5a-positive endosomes of increased size and proximity from the plasma membrane (Fig. 3, 1 h; Anti-HA). It was notable that the time-dependent internalization of the hIP observed in the immunolocalization assays paralleled that observed in the ELISA-based assays, where maximum loss of cell surface expression is achieved by 1 h. Furthermore, consistent with the latter, following 4 h stimulation with cicaprost, while the hIP was predominantly co-localized to Rab5a positive endosomes there was also evidence that some of the pre-labelled receptor had recycled back to the plasma membrane as evidenced by its increased detection at the cell surface (Fig. 3, 4h).

### 3.3 hIP-mediated activation of Rab5

Rab GTPases act as molecular switches, cycling between active GTP-bound and inactive GDP-bound states [45]. The activated form is membrane bound and, after inactivation by their specific GTPase activating protein(s) (GAPs), the GDP-bound Rab is extracted from the membrane by their specific GDP-dissociation inhibitor(s) (GDIs) and recycled back to the cytosol to await further signaling [46,47]. To address whether hIP signaling may actually lead to direct activation or engagement of Rab5, HEK.hIP cells were stimulated with cicaprost over a 4 h time course prior to their fractionation into crude membrane/particulate (P_100_) and cytosolic/soluble (S_100_) fractions to monitor agonist-induced changes in subcellular location and activation of Rab5. In the absence of agonist stimulation, there was almost a 50: 50 distribution of Rab5 protein located between the cytosol and membrane fractions (Fig. 4A, upper panels). Whilst there was no change in the overall level of Rab5 protein expression over the duration of cicaprost stimulation, there was a significant decrease in the levels of Rab5 in the S_100_ fraction as early as 30 min, which continued to decrease to barely detectable levels at 2–3 h post agonist stimulation (Figs. 4A and B). Moreover, at 4 h post-agonist stimulation there was evidence of significant Rab5 recycling back to the S_100_ fraction (Fig. 4A, upper panels; Fig. 4B). These data suggest that agonist-dependent activation of the hIP leads to direct time-dependent activation of Rab5 leading to its net translocation from the cytosolic/soluble fraction leading to its activation. Failure to detect corresponding agonist-dependent increases in Rab5 expression in the P_100_ fractions owing to its translocation from the S_100_ fractions, except at the 2 h time point, was most likely simply due to the saturation of the chemiluminescence detection system owing to the already high levels of Rab5 associated with the P_100_ fraction, even in the absence of agonist. It was also notable that the time-dependent activation/translocation of Rab5 from the S_100_ fraction almost paralleled that observed in the ELISA-based internalizations and the immunolocalizations whereby the activation of Rab5 in response to hIP signaling was both rapid (~ 30 min) and transient (~ 3 h).

Similar to Rab5, the molecular chaperone protein HDJ-2 is an isoprenylated protein that is dually located in the membrane (P_100_) and soluble/cytosolic (S_100_) fractions of the cell [48]. Hence, to further confirm the specificity of the cicaprost-induced translocation of Rab5 from the soluble fractions, we also examined the expression of HDJ-2 protein in the various subcellular fractions as a function of cicaprost stimulation by rescreening the Rab5 immunoblots with anti-HDJ-2 antibody (Fig. 4A, lower panels). HDJ-2 protein was readily detected in both the S_100_ and P_100_ fractions under basal conditions, but unlike that of the Rab5 GTPase, it did not undergo altered subcellular relocation in response to cicaprost stimulation (Fig. 4A). These data confirm the specificity of the Rab5 data and, moreover, confirm that the observed decline in Rab5 expression in the S_100_ fractions owing to its translocation from the cytosolic fractions was indeed a cicaprost-induced event and was not due to lack of uniformity in protein loading, for example.

In light of the latter evidence that endogenous Rab5 may be activated in response to cicaprost stimulation, we next sought to examine the agonist-induced co-localization of endogenous Rab5 with the hIP. As in the previous experiments, cell surface HA-tagged hIPs were pre-immunolabelled with anti-HA 101R prior to cicaprost stimulation to follow their internalization, whereas endogenous Rab5 was detected post-stimulation and fixation, under permeabilizing conditions using anti-Rab5 (S-19). The pre-labelled hIP was detected exclusively at the cell surface, as expected, and thereafter re-localized away from the plasma membrane to endocytic vesicles in a time-dependent manner in response to cicaprost (Fig. 4C, Anti-HA). Furthermore, internalization led to an almost complete loss of cell surface hIP at 2 h post cicaprost stimulation (Fig. 4C, Anti-HA, 2 h), with evidence of recycling back to the cell surface at 4 h (Fig. 4C, Anti-HA, 4 h). In the absence of agonist (Fig. 4C, Anti-Rab5, 0 h), endogenous Rab5 exhibited punctuate intracellular staining, with no evidence of co-localization with the pre-labelled cell-surface hIPs. In response to cicaprost, Rab5 positive vesicles became more pronounced and co-localized with the hIP, as observed in the overlaid image (Fig. 4C, Overlay, 2 and 4 h). Hence, these data confirm that cell surface hIPs can internalize to native Rab5-positive endosomes in response to cicaprost stimulation.

### 3.4 Co-immunoprecipitation of Rab5a with hIP

**Fig. 5:**
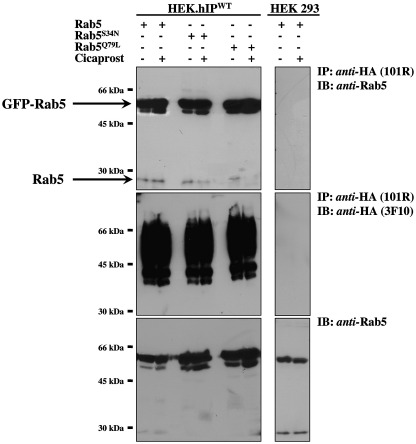
Co-immunoprecipitation of Rab5a with the hIP. HEK.hIP cells, or as controls, HEK 293 cells, transiently transfected with pEGFPCI:Rab5a, pEGFPC1:Rab5a^S34N^, pEGFPC1:Rab5a^Q79L^, were incubated with either the vehicle (−) or 1 μM cicaprost (+) at 37 °C for 2 h, prior to immunoprecipitation with anti-HA 101R antibody. Immunoprecipitates (IP) were resolved by SDS-PAGE and were either immunoblotted (IB) versus anti-Rab5 antibody (upper panels) or anti-HA 3F10-HRP antibody (middle panels), as indicated to the right of the panels. Aliquots of whole cell lysates (approx. 50 μg/lane) were also resolved by SDS-PAGE and immunoblotted with anti-Rab5 antibody (lower panels). The relative positions of the molecular size markers are indicated to the left of the panels. Results presented are representative of at least 3 independent experiments.

Taken together, data herein have established that the hIP undergoes significant time-dependent internalization in response to cicaprost stimulation; over-expression of Rab5a increases that internalization; the internalized hIP co-localizes to Rab5a positive endosomes and agonist stimulation leads to direct translocation-dependent activation of Rab5. Bearing this in mind, through a series of co-immunoprecipitations, it was next sought to investigate whether the hIP may physically/directly interact with Rab5, such as in response to agonist-stimulation. To this end, HEK.hIP cells were co-transfected with plasmids encoding GFP-tagged forms of Rab5a, Rab5a^S34N^ and Rab5a^Q79L^ and their presence in the anti-HA-hIP immunoprecipitates investigated as a function of agonist-stimulation. In the absence of agonist-stimulation, both recombinant GFP-Rab5a (55 kDa) and endogenous Rab5 (25 kDa) proteins were readily detected following immunoprecipitation of the hIP with anti-HA antibody, while no such proteins were detected in the corresponding immunoprecipitates from either the parental HEK 293 cells (Fig. 5; upper panel) or from control HEK.β-Gal cells (data not shown). Furthermore, following cicaprost stimulation, there was an increase in the amount of both GFP-Rab5a and endogenous Rab5 associated with the hIP immunoprecipitates (Fig. 5; upper panel). The increase in immunoprecipitation of Rab5a in response to agonist was not due to an increase in hIP or Rab5a expression levels (data not shown and Fig. 5; lower panel) or in the level of the hIP present in the anti-HA immunoprecipitates per se (Fig. 5; middle panel) and, hence, was due to enhanced interaction between hIP and Rab5a. Similarly, immuno-detection with anti-GFP showed that the hIP and Rab5a interaction was enhanced in response to agonist (data not shown).

Co-immunoprecipitation of the GTP-binding defective variant Rab5a^S34N^ was also observed with the hIP even in the absence of agonist (Fig. 5; upper panel). The extent of association between the hIP and Rab5a^S34N^ was similar to that of wild type Rab5a (Fig. 5; upper panel) as were the amount of hIP present in the immunoprecipitates and the levels of GFP-Rab5a and -Rab5a^S34N^ over-expression in the HEK.hIP cells (Fig. 5, middle and lower panels, respectively). Furthermore, agonist stimulation caused an increased association between the hIP and Rab5a^S34N^ (Fig. 5; upper panel). Likewise, the constitutively active, GTPase defective Rab5a^Q79L^ co-immunoprecipitated with the hIP to levels that were somewhat higher than that of the wild type Rab5a even in the absence of agonist (Fig. 5, upper panel). Furthermore, cicaprost-stimulation caused a further increase in hIP: Rab5a^Q79L^ association (Fig. 5, upper panel). Taken together, these data confirm that the hIP can physically and constitutively interact with Rab5a in the absence of agonist but that there is an increased association in response to agonist activation. Moreover, hIP:Rab5a interaction is somewhat independent of the guanine nucleotide (GDP/GTP) binding status suggesting that the Switch I and II domains of Rab5 do not greatly influence that interaction.

### 3.5 Importance of the C-tail of the hIP for agonist-induced internalization

The prostacyclin receptor (IP) is somewhat unique among the superfamily of GPCRs in that it undergoes isoprenylation, or more specifically farnesylation, within its ‘-CAAX’ motif located within its carboxyl-terminal (C)-tail domain [15,16,40,49]. Consistent with a role for isoprenylation for IP function, impairment of isoprenylation significantly reduces agonist-induced hIP internalization [16,40]. Moreover, in another study, it was found that deletion of a substantial portion of the C-tail domain (residues 312–386) generated a truncated hIP receptor, termed “C-Del” that failed to undergo short-term agonist sequestration [24]. Contrary to the latter, Haase et al., found that deletion of the terminal 68 residues did not significantly affect the ability of the hIP to undergo long-term agonist-induced internalization [39]. Hence, it is evident there is substantial conflict in the literature regarding both the mode(s) of agonist-induced internalization of the IP and, more specifically, regarding the possible requirement for and/or involvement of its C-tail domain in that internalization [16,24,39,50]. Bearing this in mind, we next sought to investigate cicaprost-induced internalization by two previously characterized truncation variants of the hIP, namely hIP^Δ312^ (devoid of the terminal residues 312–386, including the ‘-CAAX’ motif) and hIP^Δ307^ (devoid of the terminal residues 307–386, including the palmitoylated residues at Cys^308^ and Cys^311^ and the ‘-CAAX’ motif; [17]).

**Fig. 6:**
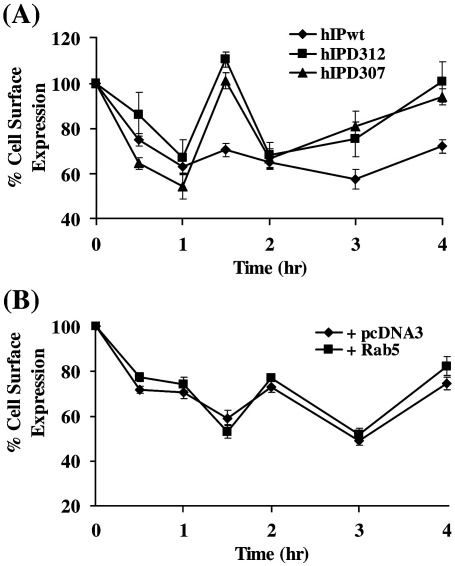
Cicaprost-induced Internalization by the hIP^Δ312^ and hIP^Δ307^. Panel A: HEK.hIP, HEK.hIP^Δ312^ and HEK.hIP^Δ307^ cells were stimulated with 1 μM cicaprost for 0–4 h at 37 °C. Panel B: HEK.hIP^Δ312^ cells transiently co-transfected with either pCMV5:Rab5a or pcDNA were stimulated with 1 μM cicaprost for 0–4 h at 37 °C. Panels A and B: expression of cell surface HA-tagged hIPs was detected by the ELISA-based assay using anti-HA 101R antibody, as described in experimental procedures. Results presented are expressed as mean cell surface expression as a percentage of that at 0 h (% cell surface expression ± S.E.M.) as a function of time (h). Results are representative of at least three independent experiments, each carried out in triplicate.

Initially, both the HEK.hIP^Δ312^ and HEK.hIP^Δ307^ cell lines [17] were confirmed to express near equivalent levels of the hIP^Δ312^ and hIP^Δ307^, respectively, as assessed by radioligand binding (Table 1) and, consistent with previous reports [16], it was confirmed that their relative affinities for [^3^H]iloprost (Kd) or maximal expression (Bmax) were not significantly different to those of the wild type hIP ([16] and data not shown). However, in examining agonist-induced internalization to monitor net changes in over-all cell surface expression, it was evident that the pattern and profile of trafficking by both the hIP^Δ312^ and hIP^Δ307^ varied considerably and were significantly different to that of the wild type hIP (Fig. 6A, *p* < 0.0001 and *p* < 0.0001, respectively; ANOVA). More specifically, the hIP, hIP^Δ312^ and hIP^Δ307^ each underwent internalization during the initial 60 min post-cicaprost stimulation. However, after 1 h, while the hIP^WT^ continued to be internalized up until the 3 h time-point, cell surface expression of both hIP^Δ312^ (*p* ≤ 0.0001) and hIP^Δ307^ (*p* ≤ 0.0001) significantly increased at 90 min, showing 100% cell surface expression, before a further decrease in cell surface expression at 2 h (Fig. 6A). By 4 h post-agonist stimulation, the hIP, hIP^Δ312^ and hIP^Δ307^ each displayed increased cell surface expression suggesting their recycling back to the cell surface, consistent with our previous findings with the hIP (Fig. 1A). It was also notable that in the case of both the hIP^Δ312^ (*p* ≤ 0.005) and hIP^Δ307^ (*p* ≤ 0.0001), there were significantly more receptors located at the cell surface at 4 h post-agonist stimulation, compared to the wild type hIP.

Hence, these ELISA data herein suggested that both the hIP^Δ312^ and hIP^Δ307^ display significantly altered patterns of trafficking to and from the plasma membrane relative to the hIP. Therefore, we next investigated whether Rab5 could promote and/or maintain the internalization of either hIP^Δ312^ or hIP^Δ307^. Over-expression of Rab5a had no significant effect on the level or general pattern of agonist-induced internalization by the hIP^Δ312^ (Fig. 6B, *p* = 0.0573; ANOVA) or the hIP^Δ307^ (data not shown) over a 4 h incubation period. It was notable there was a delay in the initial rate of internalization by the hIP^Δ312^ in the presence of exogenous Rab5a (Fig. 6B) relative to that observed in its absence (Fig. 6A), where maximum internalization was achieved at 90 min in the former (Fig. 6B), compared to 60 min for the latter (Fig. 6A). This delay was most likely due to the transfection process and/or recovery of the cells post-transfection rather than a Rab5a-mediated effect as the control pcDNA3-transfected cells exhibited a similar delay in internalization for both the hIP^Δ312^ (Fig. 6B) and hIP^Δ307^ (data not shown). Hence, unlike that of the hIP, over-expression of Rab5a did not specifically augment or alter the trafficking by the hIP^Δ312^ or hIP^Δ307^, suggesting that the C-tail domain may in someway be involved in the interaction with Rab5a.

**Fig. 7:**
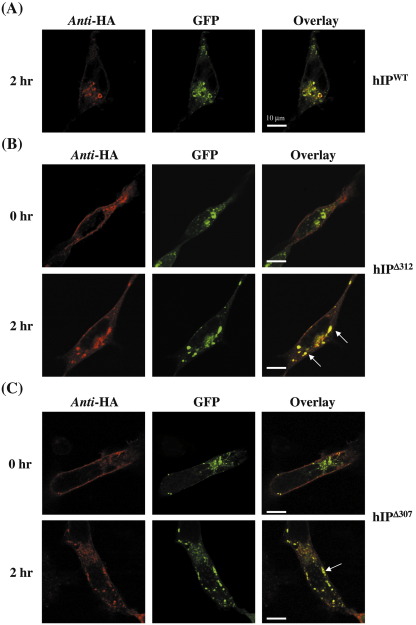
Cicaprost-induced Co-localisation of hIP^Δ312^ and hIP^Δ307^ with Rab5a. HEK.hIP (panel A), HEK.hIP^Δ307^ (panel B) or HEK.hIP^Δ312^ (panel C) cells, transiently transfected with pEGFPCI:Rab5a, were pre-labelled with anti-HA 101R primary antibody for 1 h at 4 °C; thereafter, cells were either analyzed directly (0 h) or were incubated at 37 °C for 2 h with 1 μM cicaprost (2 h), as indicated. Cells were fixed and permeabilized prior to detection of HA-tagged hIPs, with anti-mouse AlexaFluor594 conjugated secondary antibody, and enhanced GFP:Rab5a expression using Carl Zeiss Lazer Scanning System LSM510 and Zeiss LSM Imaging software. Data presented are representative images from 3 independent experiments from which at least 10 fields were viewed at ×63 magnification, where the horizontal bar represents 10 μm. The reader is referred to the web version of this article to see color images of this figure.

To further investigate the effect of the C-tail deletion on Rab5-mediated internalization of hIP, we investigated whether the hIP^Δ312^ and hIP^Δ307^ co-localize with Rab5 endosomes in response to agonist, as observed with the wild type hIP (Figs. 2 and 7A). In the absence of cicaprost, both the pre-immunolabelled hIP^Δ312^ and hIP^Δ307^ were detected at the cell surface while GFP-Rab5a exhibited punctuate intracellular staining, consistent with small endocytic vesicles with little co-localization between either hIP^Δ312^ or hIP^Δ307^ and Rab5a (Figs. 7B and C; 0 h). Whilst the hIP^Δ312^ and hIP^Δ307^ each moved from the cell surface into the small endosomal type vesicles, showing some co-localization with Rab5a in response to cicaprost stimulation (Figs. 7B and C, 2 h), it was also evident that the extent of their internalization was significantly impaired relative to the hIP. It was also noteworthy that from several independent experiments that the size of the endosomal vesicles generated in response to cicaprost stimulation in HEK.hIP^Δ312^ and HEK.hIP^Δ307^ cells were smaller and closer to the cell surface than those generated in HEK.hIP cells under similar incubation conditions (1 μM cicaprost, 2 h; Figs. 2 and 7A). In agonist-treated HEK.hIP cells the endocytic vesicles were on average 2–3 μm in diameter whilst those in HEK.hIP^Δ312^ and HEK.hIP^Δ307^ reached a maximum diameter of 1.5 μm.

**Fig. 8:**
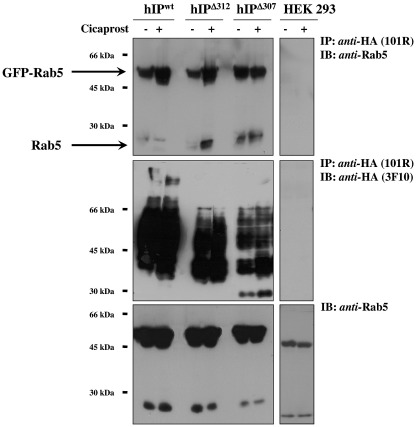
Co-immunoprecipitation of Rab5a with hIP^Δ312^ and hIP^Δ307^. HEK.hIP^Δ312^ and HEK.hIP^Δ307^ cells, or as a control, HEK 293 cells, transiently transfected with pEGFPCI:Rab5a, were incubated with either the vehicle (−) or 1 μM cicaprost (+) at 37 °C for 2 h, prior to immunoprecipitation with anti-HA 101R antibody. Immunoprecipitates (IP) were resolved by SDS-PAGE and were either immunoblotted (IB) versus anti-Rab5 antibody (upper panels) or anti-HA 3F10-HRP antibody (middle panels), as indicated to the right of the panels. Aliquots of whole cell lysates (approx. 50 μg/lane) were also resolved by SDS-PAGE and immunoblotted with anti-Rab5 antibody (lower panels). The relative positions of the molecular size markers are indicated to the left of the panels. Results presented are representative of at least 3 independent experiments.

From co-immunoprecipitation studies it was confirmed that in the absence of agonist-stimulation, both recombinant GFP-Rab5a (55 kDa) and endogenous Rab5 (25 kDa) proteins were readily detected in the anti-HA-immunoprecipitates from HEK.hIP, HEK.hIP^Δ312^ and HEK.hIP^Δ307^ cells to near comparable levels, while no such proteins were detected in the equivalent immunoprecipitates from either the parental HEK 293 cells (Fig. 8; upper panel) or from control HEK.β-Gal cells (data not shown). This hIP:Rab5a interaction was further enhanced following stimulation of HEK.hIP, HEK.hIP^Δ312^ and HEK.hIP^Δ307^ cells with 1 μM cicaprost for 2 h. These data confirm that both the wild type hIP and its truncated variants hIP^Δ312^ and hIP^Δ307^ were capable of directly interacting with Rab5a, both in an agonist-independent and -dependent manner.

### 3.6 Cicaprost-induced movement of Rab5 in EA.hy 926 cells

**Fig. 9:**
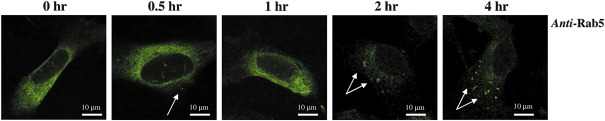
Cicaprost-induced re-location of Rab5 in EA.hy 926 cells. EA.hy 926 cells were stimulated with vehicle or 1 μM cicaprost at 37 °C for 0–4 h. Cells were fixed and permeabilized prior to detection of endogenous expression of Rab5 with rabbit anti-Rab5 and anti-rabbit AlexaFluor488 conjugated secondary antibodies expression using a Carl Zeiss Lazer Scanning System LSM510 and Zeiss LSM Imaging software. Data presented are representative images from 3 independent experiments from which at least 10 fields were viewed at ×63 magnification, where the horizontal bar represents 10 μm. The reader is referred to the web version of this article to see color images of this figure, where relevant.

In order to further investigate the involvement of Rab5 in the internalization of the hIP, we also examined changes in the localization of Rab5 endogenously expressed in endothelial cells, namely in the human endothelial EA.hy 926 cell line [44], in response to stimulation of endogenous hIPs with cicaprost. In the absence of agonist, Rab5 exhibited punctuate staining, with little evidence of its expression at the cell surface (Fig. 9, 0 h). In response to cicaprost-stimulation, the Rab5-positive vesicles become substantially more pronounced in a time-dependent manner such that at 2 h post-stimulation, they coalesced into fewer but significantly larger vesicles (Fig. 9, 2 and 4 h). These data in endothelial EA.hy 926 cells provide further independent evidence of the role of Rab5 in agonist-dependent internalization of the hIP.

## 4 Discussion

A critical feature of the GPCR signaling paradigm is desensitization, the process that regulates the signal in response to temporary or sustained agonist stimulation [18,19]. Rapid desensitization is achieved by receptor phosphorylation such as by second messenger-dependent protein kinase (PK) A or PKC or by GRKs [30,51]. Another means by which GPCRs are critically regulated post-agonist activation is sequestration or internalization of the GPCR from the plasma membrane into the various intracellular compartments making them unavailable for further stimulation of the primary signaling path, at least. The best characterized mechanism of GPCR internalization is the clathrin-mediated endocytic pathway where, classically, GRK phosphorylation of the agonist-engaged GPCR leads to the recruitment of β-arrestin(s) which, in turn, act as intermediary adaptor proteins targeting the receptor to clathrin coated vesicles [38,52–54]. However, it is well recognized that β-arrestin-mediated internalization of GPCRs via CCVs may represent only one of the many pathways leading to GPCR endocytosis [55–57].

The human prostacyclin receptor (hIP) has been shown to undergo rapid agonist-induced, PKC-phosphorylation and desensitization of signaling in human platelets and other cell types [20–24]. Furthermore, it undergoes rapid agonist-induced internalization by, as yet, largely unknown mechanism(s) that are independent of PKC and the classical GRK/β-arrestin pathway [24,58]. In the current study and consistent with previous reports [24,58], through ELISA-based internalization assays it was confirmed that hIP internalizes in response to its agonist cicaprost in a time- and concentration-dependent manner. From the time course assays, it was evident that most of the internalization occurred during the initial 60 min incubation period, with some 30–40% of the total hIP on the cell surface undergoing internalization. Thereafter, the rate of internalization declined such that the overall level of internalized hIP remained at approximately 40% for a duration of 3 h post-agonist stimulation while at 4 h, some 50% of the internalized hIP recycled to the cell surface. On prolonged cicaprost stimulation (4–8 h), we observed that some 50% of the internalized hIP failed to recycle back to the plasma membrane (data not shown) and, therefore, reasonably assume that it underwent agonist-induced turnover/degradation such as through onward trafficking of the internalized hIP to lysosomes.

However, as the ELISA-based assay is an end-point assay and can only measure net changes in cell surface expression, we also investigated whether the hIP may undergo agonist-induced localization to Rab5 endosomes through more direct imaging based-studies. Through confocal microscopy, it was demonstrated that the agonist-stimulated hIP moves from the plasma membrane into large intracellular punctuate type vesicles, reminiscent of CCVs or endocytic vesicles. Alternative mediators of GPCR internalization and indeed trafficking have been identified including the Rab GTPases [30,59]. Rab proteins are the largest subgroup of the Ras-like small GTPases with at least 60 members identified in humans and act as molecular switches to regulate and facilitate numerous cellular processes such as endocytosis, intracellular vesicle transport and exocytosis [30,60,61]. Rab5 is involved in clathrin-mediated endocytosis where it is recruited to the endocytic vesicles, sequestering ligands into clathrin-coated pits and directing early endosome fusion [30,59,61]. Hence, herein, in view of the agonist-induced internalization coupled with the immunolocalization data, it was sought to determine whether Rab5 had a role in internalization of the hIP. Through ELISA-based assays, it was established that over-expression of Rab5a led to a significant increase in cicaprost-induced internalization throughout the initial 3 h duration post-agonist stimulation but did not interfere with the level of the hIP recycled at the 4 h time point. Furthermore, using GFP-tagged Rab5a, it was clearly established that the hIP internalizes to and co-localizes with Rab5a in intracellular endocytic vesicles in response to agonist stimulation. In contrast to that observed with certain other GPCRs [33,35], over-expression of Rab5a^S34N^ did not appear to substantially reduce cicaprost-induced internalization of the hIP when assessed through the ELISA-based internalization assays. This may be due to the high levels of endogenous Rab5 present in HEK 293 cell line itself, as confirmed by immunoblotting. However, contrary to the latter data, confocal microscopy showed that, in the presence of Rab5a^S34N^, agonist-internalization of the hIP differed significantly from that observed with the wild type Rab5a. In the presence of Rab5a^S34N^, the extent of relocalization of the hIP away from the plasma membrane was significantly impaired and the pattern of intracellular staining was more diffuse, lacking the distinct vesicular pattern typically associated with Rab5-positive endosomes. On the other hand, in the presence of constitutively active Rab5a^Q79L^, agonist-induced internalization was significantly increased and the internalized hIP co-localized to substantially enlarged Rab5a^Q79L^ positive endosomes. Taken together, these data strongly suggest that Rab5a has an active role in the internalization of the hIP and is necessary for its efficient sorting to Rab5a-positive endosomes.

Rab GTPases cycle between active GTP-bound and inactive GDP-bound states, controlled by guanine nucleotide exchange factors (GEFs) and GTPase activating factors (GAPs) [61,62]. Membrane bound Rabs are active and, after inactivation by their specific GAPs, the GDP-bound Rabs become extracted from the membrane by GDP-dissociation inhibitor (GDI) where they remain complexed, in their GDP-bound state, ready for the next cycle of activation and recruitment to the membrane [61,62]. Herein, through subcellular fractionation studies, there was a substantial, time-dependent decrease in the levels of Rab5 in the soluble fraction for the initial 3 h post-agonist stimulation while at 4 h, there was evidence of significant Rab5 recycling back to the cytosolic/S_100_ fraction. These data suggest that agonist-activation of the hIP may directly couple to Rab5, promoting the release of GDI and, in turn, stimulating membrane association and GDP/GTP exchange to regulate hIP trafficking from the cell surface to Rab5-positive endosomes. Furthermore, these data also suggested a direct association between the hIP and Rab5. Through co-immunoprecipitations, a direct physical interaction between hIP and Rab5a was confirmed even in the absence of agonist but which was enhanced in response to cicaprost stimulation, consistent with the cicaprost-induced translocation of Rab5 from the soluble/cytosolic fraction. The reason for an apparent discrepancy between the co-immunoprecipitation and co-localization data is due to the way in which the two types of experiments were performed. To monitor the role of Rab5 in agonist-induced internalization of the hIP by confocal microscopy, cell surface hIPs were pre-immunolabelled prior to agonist-stimulation. In response to agonist, only changes in the subcellular localization, and subsequent colocalization with Rab5a, of pre-labelled cell surface hIP expression was monitored. Hence, in unstimulated cells, lack of co-localization of Rab5a with the hIP is due to the fact that only hIPs located at the cell surface were labelled/detected. On the other hand, in the co-immunoprecipitations, all HA-tagged hIPs (both cell surface and intracellular) were immunoprecipitated and hence, as is suggested from the co-immunoprecipitations, Rab5 associates with the hIP even in the absence of agonist. Moreover, through further co-immunoprecipitations, it was established that similar to the wild type Rab5a, both Rab5a^S34N^ and Rab5a^Q79L^ are capable of interacting with the hIP and that such interactions were enhanced on agonist-stimulation. Taken together, these data clearly suggest that GTP-binding or hydrolysis may not be a limiting factor for Rab5:hIP complex formation but from immunolocalizations involving Rab5a^S34N^, GTP-loading is necessary to drive endosome formation and hIP sorting to such endosomes. Moreover, the translocation assays and the confocal imaging data suggest that the hIP is not simply passive cargo in the endocytosis process but that it directly influences the subcellular localization and association of Rab5 with the endosomal membranes. Consistent with that hypothesis, stimulation of endogenous hIPs expressed in the endothelial EA.hy 926 cell line with cicaprost led to a profound, time-dependent relocalization of endogenous Rab5 to enlarged endocytic vesicles.

The GTPase dynamin mediates the first steps of endosome formation at sites of clathrin-coated vesicles (CCVs) and caveoli [25,26]. Consistent with this, over-expression of a dominant negative form of dynamin, namely Dyn^K44A^, partially impaired agonist-internalization of the hIP [24]. Herein, while expression of Dyn^K44A^ or Rab5a^S34N^ partially impaired cicaprost-induced hIP internalization, co-expression of both factors together did not lead to further impairments suggesting that dynamin and Rab5 regulate hIP internalization through a common pathway rather than through distinct mechanisms.

The internalization of numerous GPCRs has been found to be dependent on the presence of an intact C-tail domain [63,64]. As stated, in the case of the hIP, there is conflicting evidence regarding the requirement of the C-tail domain for internalization [16,24,39]. It is reasonable to speculate that the conflicting data regarding the C-tail may be attributable to differences between the cell types employed and the complement of proteins that make up their endocytic machinery, as has been suggested for other receptors [65]. Hence, herein, we sought to clarify the requirement for the C-tail in hIP internalization in HEK 293 cells. As stated, the C-tail of the hIP is complex being subject to both palmitoylation, at Cys^308^ and Cys^311^, and farnesylation, at Cys^383^ [16,17]. Using previously characterized deletion variants hIP^Δ312^ and hIP^Δ307^, in keeping with the findings of Smyth et al. [24] cicaprost-induced internalization by the hIP^Δ312^ or hIP^Δ307^did not differ substantially to that of the wild type hIP within the first 60 min post-agonist stimulation. However, after 60 min, cell surface expression of the truncated hIP^Δ312^ and hIP^Δ307^ variants differed significantly from the hIP with evidence of altered trafficking or recycling, i.e., they exhibited altered time-dependent movement to and from the cell surface followed by further internalization, and after 4 h incubation both hIP^Δ312^ and hIP^Δ307^ were located at the cell surface. The time-dependent variability in the internalization and cell surface expression of the hIP^Δ312^ and/or hIP^Δ307^ may explain the apparent conflict in data between the previously reported research studies [24,39]. Moreover, the data herein suggests that while the C-tail domain of the hIP may not be essential for internalization per se, it does appear to be required to maintain the internalized receptor within the endocytic pool, preventing it from recycling back to the plasma membrane, such as occurs in the case of the hIP^Δ312^ and hIP^Δ307^ at 90 min. In contrast to that which occurred for the wild type hIP, over-expression of Rab5a did not influence the internalization pattern of the hIP^Δ312^ or hIP^Δ307^suggesting that the C-tail may actually be involved in or required for the Rab5 interaction. Co-immunoprecipitations categorically established that both the hIP^Δ312^ or hIP^Δ307^ are capable of directly interacting with Rab5a and that, similar to the hIP, such interactions are enhanced in response to cicaprost stimulation. However, whilst deletion of the C-tail did not inhibit interaction with Rab5a, confocal imaging established that the truncated hIP^Δ312^ or hIP^Δ307^ had significantly impaired internalization and co-localization to Rab5a-positive endosomes. Specifically, in contrast to the distinct enlarged ring-like vesicles observed with the internalized wild type hIP, such as at 2 h post-agonist stimulation, the hIP^Δ307^ and hIP^Δ312^ were found associated with significantly smaller Rab5a positive vesicles that were located much closer to the plasma membrane, suggestive of impaired internalization or subsequent trafficking. Taken together, these data generated with the hIP^Δ312^ and hIP^Δ307^ suggests that while the C-tail is not essential for Rab5a interaction, it appears to have a role in regulating further trafficking of the hIP from the Rab5-early endosomes, perhaps to other Rab-containing transport vesicles, preventing it from recycling to the plasma membrane in a dysregulated manner.

Rab5 can play quite diverse roles in mediating agonist-induced internalization of various GPCRs. In the case of the dopamine D2 receptor, internalization was accelerated by over-expression of Rab5a and Rab5a^Q79L^ and inhibited by Rab5a^S34N^ [35]. β_2_AR internalization was also inhibited by Rab5a^S34N^, preventing receptor dephosphorylation and resensitization [33]. Internalization of the neurokinin-1 receptor (NK1R) involves dynamin- and β-arrestin-dependent processes, followed by Rab5a-mediated transport from early endosomes to Rab4a- and Rab11a-containing recycling endosomes [66]. In this case, over-expression of either Rab5a or its dominant negative variant did not affect receptor internalization per se but influenced the ability of the NK1R to translocate from superficial vesicles beneath the plasma membrane into intracellular endosomes. Likewise, agonist-mediated internalization of the angiotensin II type 1A receptor (AT_1A_R) was not affected by Rab5 protein expression despite the formation of Rab5-AT_1A_R protein complexes mediated, at least in part, by the last 10 amino acid residues of the C-tail of AT_1A_R itself [37].

Whilst the latter studies clearly indicate an involvement of Rab5 in internalization/trafficking of certain GPCRs, in the broader context of the superfamily, to date it is in no way a widely recognized phenomenon or indeed predictable. More particularly, this is the first study showing a role for Rab5 in the internalization/trafficking of the prostacyclin receptor. In order to explain the role of Rab5 in the internalization of the hIP, we propose that on agonist stimulation, the hIP is initially recruited to CCVs at the plasma membrane. The involvement of clathrin-coated pits has been confirmed whereby concanavalin A and sucrose, inhibitors of clathrin-mediated trafficking, impaired agonist-induced internalization of the hIP in HEK 293 cells [24]. Thereafter, a proportion of the hIPs are internalized via a dynamin- and Rab5-dependent mechanism. Confocal imaging of HEK.hIP cells over-expressing the GFP-tagged Rab5a demonstrated that there was rapid co-localization of the hIP to Rab5a positive vesicles at or just below the cell surface indicating that Rab5a affects internalization of the hIP at an early stage. Cell fractionation data herein also demonstrate that there is significant and rapid translocation of Rab5 from the soluble to particulate/membrane fraction in response to cicaprost-stimulation and, as assessed through confocal imaging, there was cicaprost-dependent relocalization of endogenous Rab5 in EA.hy 926 cells. Whilst multiple roles for Rab5a in endosome formation and fusion have been identified, the role of Rab5a at the plasma membrane is being increasingly understood [59]. From studies involving the hIP^Δ312^ and hIP^Δ307^ variants, it appears that while the C-tail of the hIP is not absolutely essential for the Rab5-mediated internalization, it has a role in the onward trafficking of the hIP. This suggests that there are sequences/domains within the C-tail of the hIP that regulate the subsequent fate of the internalized receptor. Once conveyed to Rab5-containing endosomes, we propose that the internalized hIP is sorted and subsequently either recycled back to the cell surface via recycling endosomes or transported to lysosomes for degradation. These processes are most likely facilitated by other members of the Rab GTPase family, such as by sorting to Rab4 containing early endosomes and/or to Rab11 containing late endosomes and in their subsequent regulation of recycling or to Rab7 containing late endosomes and transported to lysosomes. Through follow up studies, we are currently investigating the role of Rab4 and Rab11 in post-endocytic sorting of the hIP. Moreover, we are also seeking to identify the structural determinants on the hIP and on Rab5a that mediate their specific interaction.

While the data presented herein provide compelling evidence for a critical role for Rab5 in agonist-induced internalization of the hIP, they do not exclude the possibility of other internalization pathway(s) being involved, such as via the novel cGMP phosphodiesterase 6δ (PDE6δ)-dependent pathway recently proposed by Wilson and Smyth [67]. In this current study, we determined that the hIP is subject to agonist-induced internalization mediated, at least in part, via a dynamin- and Rab5a-dependent route, requiring GDP/GTP exchange on Rab5a for hIP trafficking to endosomes. Furthermore, we have demonstrated that there is a direct interaction between the hIP and Rab5a. Our studies reveal that the C-tail is not required for this interaction or hIP internalization per se but suggest that there are determining factors within the C-tail that regulate the subsequent fate of the internalized hIP, such as during its onward agonist-induced movement to recycling endosomes, via Rab4 or Rab11, or to lysosomes for degradation. The results herein add significantly to our current understanding of the events regulating the hIP post-agonist stimulation. In view of the ever-increasing recognition of the essential role of prostacyclin and its receptor for vascular integrity and in counter-balancing/off-setting various types of vascular diseases including thrombosis, systemic hypertension, stroke and myocardial infarction [5–8], these studies provide important mechanistic insights into how hIP, and/or indeed Rab5, dysfunction may contribute to such disease processes.

## Figures and Tables

**Table 1:** Radioligand binding data

Cell type	[3H]iloprost bound (pmol/mg protein ± S.E.M; *n* = 4)
HEK.hIP^WT^	1.6 ± 0.7
HEK.hIP^Δ312^	1.4 ± 0.3
HEK.hIP^Δ307^	1.0 ± 0.2

Radioligand binding assays were carried out on HEK 293 cells stably over-expressing HA-tagged forms of hIP, hIP^Δ307^ and hIP^Δ312^ using the IP agonist [^3^H]iloprost (4 nM; 15.3 Ci/mmol) and 75 μg of whole cell protein/assay.
